# Late Pleistocene/Early Holocene Evidence of Prostatic Stones at Al Khiday Cemetery, Central Sudan

**DOI:** 10.1371/journal.pone.0169524

**Published:** 2017-01-25

**Authors:** Donatella Usai, Lara Maritan, Gregorio Dal Sasso, Gilberto Artioli, Sandro Salvatori, Tina Jakob, Tiziana Salviato

**Affiliations:** 1 Centro Studi Sudanesi e Sub-Sahariani (CSSeS), Treviso, Italy; 2 Department of Geosciences, University of Padova, Padova, Italy; 3 Department of Archaeology, Durham University, Durham, United Kingdom; 4 Department of Pathology, University of Trieste, Trieste, Italy; Leibniz-Institut fur Pflanzengenetik und Kulturpflanzenforschung Gatersleben, GERMANY

## Abstract

The recovery of three stone-like ovoid objects within the burial of a pre-Mesolithic (Late Pleistocene/Early Holocene) individual at Al Khiday cemetery (Central Sudan) raises the question of the nature and origin of these objects. The position in which the objects were found in relation to the human skeleton suggested a pathological condition affecting the individual, possibly urinary bladder, kidney stones or gallstones. To solve this issue, a multi-analytical approach, consisting of tomographic, microstructural and compositional analyses, was therefore performed. Based on their microstructure and mineralogical composition, consisting of hydroxylapatite and whitlockite, the investigated stones were identified as primary (endogenous) prostatic calculi. In addition, the occurrence of bacterial imprints also indicates on-going infectious processes in the individual. This discovery of the earliest known case of lithiasis extends the appearance of prostatic stones into the Late Pleistocene/Early Holocene, a disease which therefore can no longer be considered exclusive to the modern era, but which also affected prehistoric individuals, whose lifestyle and diet were significantly different to our own.

## Introduction

Lithiasis has affected men and women since well before the earliest written Babylonian and Egyptian reports [1,2,3]. It occurs most commonly in the gallbladder, kidney and lower urinary tract and, although it is occasionally asymptomatic, more often the condition is extremely painful. The great humanist Michel de Montaigne described his own experience with the heartfelt words “*What is this* [torture by the stone] *but testing of death again and again*? *Deliver me*, *if possible*, *from his evil which is in me*” [1].

**Fig 1:**
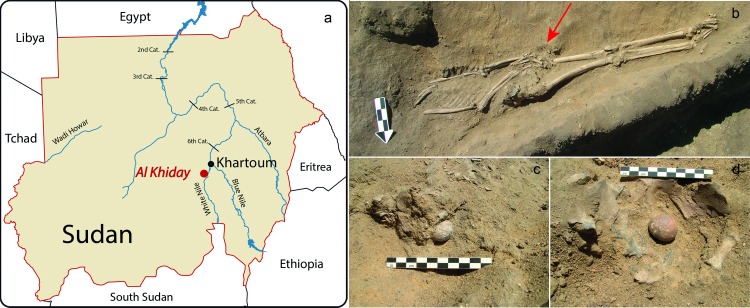
Calculi from Grave 188 at Al Khiday. a) geographic location of Al Khiday sites in Sudan; b) Pre-Mesolithic Grave 188; c-d) calculi *in situ*.

**Fig 2:**
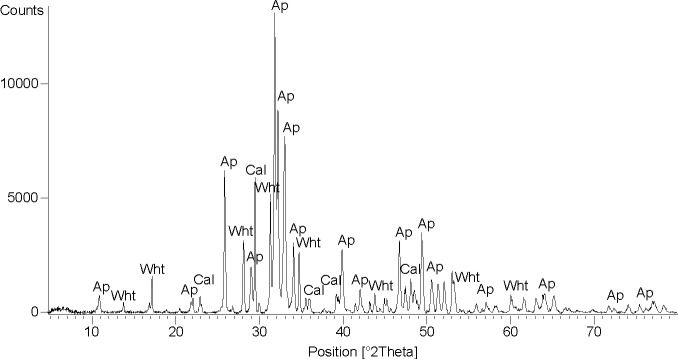
Mineralogical composition. X-ray diffraction pattern of stone S3: phase labels: Ap: apatite, Wht: whitlockite, Cal: calcite.

To date, the most ancient evidence of “calculi” comes from a Mesolithic grave excavated at the Uzzo Cave in Sicily, Italy, from the mid-seventh millennium BC [4]. A new, possibly older ‘*patient’*, has recently been discovered at the Al Khiday cemetery in Central Sudan [5,6] (Fig 1), where three stones were found in the pelvic area and near the lumbar vertebrae of an adult male burial (Fig 2). The identification of such stones on a macroscopic basis is problematic and a range of pathological calcifications and naturally occurring stones had to be considered. The possibility that these stones could have been other than pathological, for example, “sling stones” placed as offerings in the grave, was not considered because of their bulk density.

Among the many pathological origins that could possibly be attributed to these stones, gallbladder, kidney or urinary bladder stones, the first was rejected as in the absence of any disturbance of the burial prior to excavation, it would have been impossible for stones to move from the gallbladder to the lower abdomen. The two most likely candidates were kidney stones, which could enter the urethra and potentially arrive in the pelvic area, and urinary bladder stones, which appeared to be the closest alternative explanation due to their location and formal aspect. As pathological stones have differential mineralogical compositions [1], a range of analyses was carried out to determine their origin.

The cemetery of Al Khiday (Fig 1A), where the individual with pathological stones was found, is located in Central Sudan on the left bank of the White Nile, approximately 20 km south of Omdurman (Khartoum), where the White and Blue Niles meet [5,6,7,8]. One hundred and ninety graves dating to three chronological periods (pre-Mesolithic, Neolithic, and Classic/Late Meroitic) have been recovered. The oldest burials, labelled as pre-Mesolithic in date, are of interest in this context. They are the most numerous with 94 individuals and also the most interesting and intriguing population as they were buried according to a funerary rite never attested in such a high frequency in any other known prehistoric cemetery worldwide: the majority of the individuals (94%) [5,6] were buried in an extended position, lying on their front (prone). The only other known occurrence in the Nile valley is the single prone skeleton of Wadi Kubbaniya, possibly dating to the Late Palaeolithic period [9].

The pre-Mesolithic burials at Al Khiday could not be directly radiocarbon dated since the bones are completely depleted of collagen, and the tentative radiocarbon dating of the carbonate contained in hydroxylapatite also proved unsuccessful because of the multiple complex diagenetic processes affecting the bones [10].

Thus, their chronological attribution is derived by indirect inference:

1. Stratigraphic observations: more than 14 pre-Mesolithic individuals are cut by Mesolithic functional pits [5], related to the use of the area as a settlement in a period firmly dated from 6750 to 6300 cal. BC. The pre-Mesolithic burials must therefore be older than the Mesolithic period. The later Neolithic burials (n = 25) date to the mid 5^th^ millennium BC, while the Classic/Late Meroitic graves (n = 42) date to the end of the 1^st^ millennium BC/beginning of the 1^st^ millennium AD) [6];
2. Diagenesis of bones: the study of the detailed sequence of mineral deposition in the pre-Mesolithic bones [10] as well as stable isotope analyses [11] indicate that the rate of accumulation of secondary calcite in these bones is congruent with the wet climatic period occurring between the very Late Pleistocene and the very Early Holocene period [12]. Radiocarbon dating of the carbonate concretions permeating the pre-Mesolithic bones and the surrounding sediments at Al Khiday [13] indicates that the pre-Mesolithic burials must pre-date the deposition of the carbonate in the wet period 12700–11100 BC.

The aim of this work is therefore to investigate, using a multi-analytical approach, the nature and origin of these stones.

## Materials and Methods

In 2013, the pre-Mesolithic Grave 188 of the Al Khiday cemetery/settlement complex was excavated (Fig 1B–1D), which included three intriguing ovoid objects, the macroscopic features of which are consistent with those of pathological stones (Fig 2). One was found between the pelvic bones (S1) and two close to the lumbar vertebrae (S2-S3). Stone S3 was accidentally broken during the excavation. Individual 188 was buried prone, lying on the left side with the head pointing East and facing South. The skull was damaged by the digging of a later Mesolithic pit, though most of the postcranial bone fragments were preserved. Based on the morphological features of the pelvic bones the individual was an adult male, although it was impossible to establish a precise age. The individual had sustained a fracture of the left distal ulna that had healed well without any angulation or shortening of the bone. In addition, there was evidence for degenerative joint disease in all of the preserved large and small joints of the body, including the shoulders, elbows, wrists and hands, costovertebral joints, hips, as well as the left knee and foot. The three stones of grave 188, named S1, S2 and S3, were analysed using a multi-analytical approach, consisting of microstructural and mineralogical analysis. Moreover, since stone S3 was broken, it was subjected to a more detailed microstructural study.

The recovered stones show an oval, irregular shape with diameters ranging between 26 and 30 mm, weighing between 12 and 15 g (S1–S3 Figs).

The internal microstructure of the two undamaged stones, stone S1 and stone S2, was investigated through X-ray computed micro-tomography (μ-CT) and the crystallographic composition was determined by non-invasive time-of-flight neutron powder diffraction (ToF-NPD). X-ray micro-tomographic scans were performed at the Department of Geosciences (University of Padua, Italy) using a Skyscan 1172 high-resolution X-mCT scanner (Bruker). The samples were irradiated with a polychromatic X-ray cone beam, filtered with 0.5 mm aluminium foil. The X-ray source, equipped with a tungsten anode, operated at an accelerating voltage of 100 kV and a current of 100μA. The selected experimental setup ensured an appropriate compromise between X-ray transmission and absorption contrast. For each sample, 720 radiographs were acquired over a rotation of 360° with a step of 0.5° and an exposure time of 1000 ms for each projection. Three-dimensional assemblages of cross-sectional slices were obtained by tomographic reconstruction using a filtered back-projection algorithm, with a final voxel resolution of 27 μm^3^.

Mineralogical analysis was performed by X-ray powder diffraction (XRPD) on the powders produced from grinding a fragment of the broken stone S3 and on that obtained from the micro-drilling of a small portion of the complete stones (stone S1 and stone S2). The finely ground powders were measured using an X’Pert PRO diffractometer (PANalytical) in Bragg–Brentano geometry, equipped with a Cu-anode X-ray tube (40 kV and 40 mA) and a X’Celerator detector. Diffractograms were acquired in the 3°-80° 2θ range, with a step size of 0.02° and scanning time corresponding to 1 s counting time per step. The quantitative analysis was performed by Rietveld refinement, using the MAUD program [14]. Instrumental contribution to line broadening was determined by measuring the NIST Si 640c standard sample, measured using the same experimental conditions. The structural models used for the identified mineralogical phases were those provided by reference [15,16,17] for hydroxylapatite, whitlockite and calcite, respectively. A polynomial function with 6 parameters was used to describe the background, and a pseudo-Voigt function was adopted to model the diffraction peak profiles. A scale factor for each phase, background parameters, isotropic atomic displacement parameters (maintained equal for all atoms in each structure) and unit cell parameters were refined during the least squares minimization procedure. Site occupancy and atomic coordinates remained fixed during refinement for all phases. An isotropic model for the crystallite-size contribution to line broadening was applied to all phases. The quality of the Rietveld refinement was monitored by the agreement factor (Rwp < 7%).

Moreover, the non-invasive ToF-NPD diffraction measurements on stone S2 were performed at the Italian Neutron Experimental Station (INES) at the ISIS Facility (UK), by using the time of flight (ToF) technique. The multipurpose neutron diffractometer at INES is characterized by an excellent resolution (d/d = 0.1%) in the 0.1–2.1 Å range, whilst the total d-spacing range extends up to 12 Å, providing a good coverage of the kinematical range [18]. The diffractometer is equipped with 144 squashed ^3^He detectors covering 2θ angles from 11 to 170° in the horizontal scattering plane. The detectors are grouped into 9 banks, each bank composed of 16 detectors, and lie on a circle of 1 m radius from the diffractometer centre. The interval of d-spacing (or momentum transfer Q) that is available for each detector is determined for each fixed scattering angle 2θ as a function of neutron time-of-flight. The exact path distance of the neutron beam was calibrated by Rietveld refinement of the international NIST Si 640c standard sample. The collected patterns were analyzed by the Rietveld method, using the GSAS package [19]. A pseudo Voigt convoluted with a back-to-back exponential function was used for modelling the diffraction profiles; the Lorentzian (γ) and Gaussian (σ) components of the FWHM were parameterized as follows γ = γ_0_+γ_1_d +γ_2_d^2^ and σ^2^ = σ_0_^2^+σ_1_^2^d^2^+σ_2_^2^d^4^. The σ_1_ and γ_2_ terms, related to the strain and to the crystallite size respectively, were allowed to vary during the reference refinement. The scattering lengths of the structural atoms from the GSAS database were used for the refinements. The quality of the Rietveld refinement was monitored by the agreement factor (Rwp < 3%).

Microstructural analysis was performed on some fragments of the broken stone S3 by scanning electron microscopy (SEM) using a JSM Jeol 6490 equipped with a tungsten electron source and an EDAX energy dispersive X-ray detector (EDS) for micro-chemical analysis, operating at an electric potential difference of 20 kV, with a working distance between 9 and 11 mm. A small portion of the stone was also analysed by SEM without any preparation (but gold–coated), in order to perform secondary electron images (SEM-SE) and have morphological information on the crystals constituting the stone. Moreover, a fragment of stone S3 was embedded under vacuum in epoxy resin (Araldite 2020), cross sectioned, polished and studied by reflected light optical microscopy (RL-OM) and by SEM (back scattered electron images SEM-BSE).

All necessary permits were obtained for the described study, which complied all the relevant regulations. Excavation and export permits for study were provided by the National Corporation of Antiquities and Museums, Republic of the Sudan, Ministry of Tourism Antiquities and Wildlife. The project has annual rolling permits for exporting all materials to be studied and analysed (permit number: NCAM/4/B). The studied stones, inventoried as “Collezione Al Khiday–Grave 188 S1, Grave 188 S2 and Grave 188 S3”, are deposited at the Museum of Anthropology, University of Padova (Italy) and are available upon request.

## Results

The analyses performed on the stones revealed that mineralogically they are all composed of hydroxylapatite (Ap: Ca_10_(PO_4_)_6_(OH)_2_), whitlockite (Wht: Ca_9_(Mg,Fe)(PO_4_)_6_(PO_3_OH)) and minor calcite (Cal: CaCO_3_) (Fig 2), although the proportion between the mineral phases varies slightly among samples (stone S3: Ap = 75 wt%, Wht = 18 wt%, Cal = 7 wt%; stone S2: Ap = 81 wt%, Wht = 13 wt%, Cal = 6 wt%).

**Fig 3:**
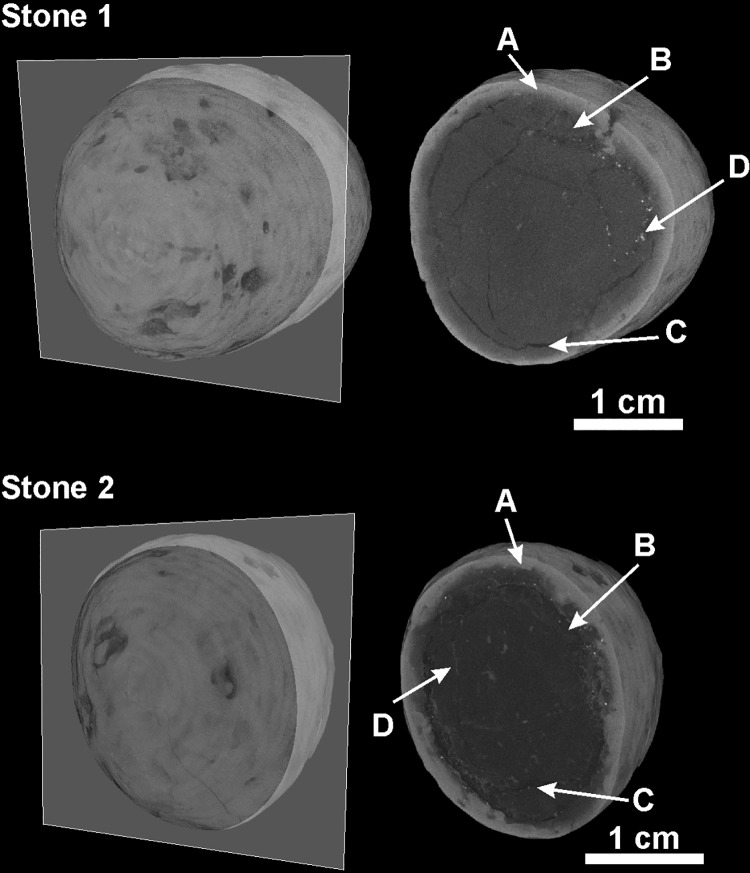
Internal structure. μ-CT reconstructed sections of stones S1 and S2: A: lighter grey colour, outermost portions; B: darker colour, innermost portions; C: black colour, fractures within the stone; D: light grey colour, calcite filling in some of the fractures.

The μ-CT reconstructed sections of stones S1 and S2 show a heterogeneous internal structure, with an outermost portion characterized by higher absorbance (A in Fig 3) with respect to the inner part (B in Fig 3), which is locally fractured (C in Fig 3).

**Fig 4:**
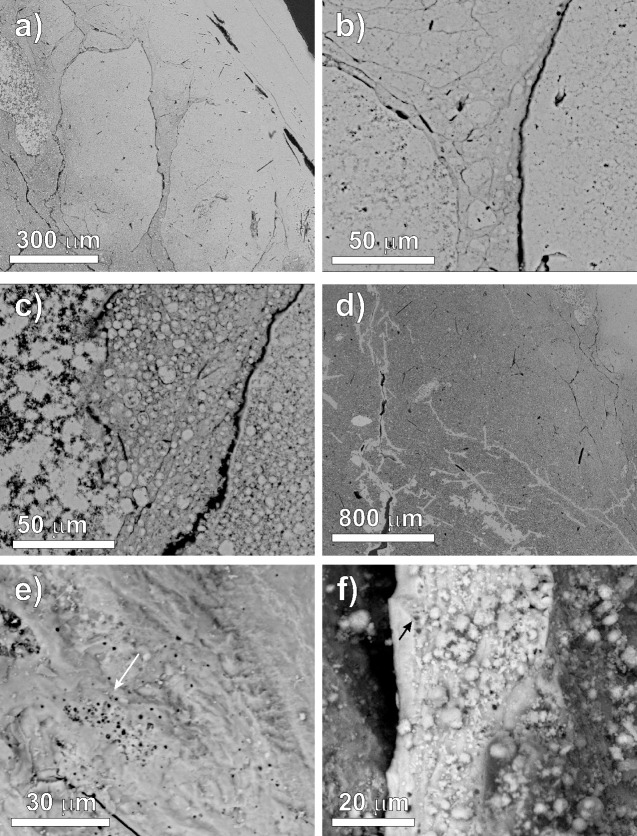
Calculi structure. SEM backscattered electron images of the microstructure of stone S3: a) outermost portion of the stone, where ovoidal structures occur; b) detail of the ovoids surrounded by a thin shrinkage rim; c) detail of the dense packing of crystals which form micron-sized spheres in some cases with a geode type structure and (on the left) acicular crystals aggregate of apatite; d) micro-fractures crossing the stone, filled by secondary calcite; e-f) micro-pores due to bacterial attack.

**Fig 5:**
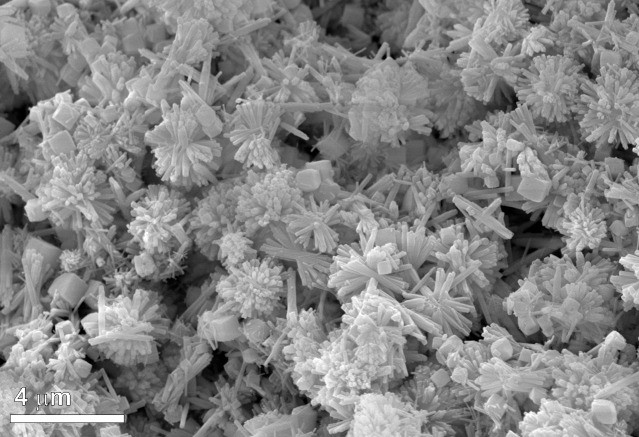
Calculus internal portion and mineral phases. SEM secondary electrons image of loose material from the internal portion of stone S3: coexisting crystals of apatite (tabular/prismatic crystals of hydroxylapatite forming radiating clusters) and whitlockite (rhombohedral crystals).

A similar structure can be observed in more detail by SEM images in stone S3, where the compact outermost portion, about 200 μm thick, gradually becomes more porous towards the core (Fig 4A). Underneath the external cortex, sub-rounded and ovoid structures measuring hundreds of micrometres are observed (Fig 4A and 4B), systematically surrounded by a thin shrinkage rim and characterized by a dense packing of about ten micron-sized spheres (Fig 4C). Micro-fractures, some filled in with calcite (Fig 4D), are also visible. In the compact portion, micro-pores (Fig 4E), in some cases surrounded by a hyper-mineralised rim (Fig 4F), due to bacterial activity, can also be observed. Moving towards the core, where the structure is less compact, the small spheres are composed of acicular crystals with a radial disposition or in some cases showing a geode-type crystallization (Fig 4C), and a chemical composition corresponding to that of hydroxylapatite. High resolution SEM secondary electron (SE) images of this porous portion show radiating aggregates of tabular/prismatic crystals of hydroxylapatite associated with rhombohedral crystals of whitlockite, about 1 μm in size (Fig 5). No traces of iron were detected by point SEM-EDS analysis on the whitlockite crystals.

Calcite fills in the micro-fractures within the stones (Figs 3 and 4D), and this can be interpreted as being precipitated during post-depositional processes. Because of the calcite distribution, its crystallization after oxalate (CaC_2_O_4_) can be safely excluded. The original mineralogical composition of the stones is therefore a mixture of hydroxylapatite and whitlockite with varying proportions. Mn, Fe-oxides/hydroxides are observed as dendrites through the optical microscope (S4 Fig) and as dark stains on the stone surface (Fig 2). Mn, Fe-oxides/hydroxides, together with calcite, are the secondary mineral phases testifying the complex diagenesis of the pre-Mesolithic individuals at the Al Khiday site [10,13].

## Discussion

The remarkable discovery of these three stones in a pre-Mesolithic individual at the Al Khiday site provided a unique opportunity to determine their nature and origin.

Firstly, a possible geogenic origin of these stones was considered, as hydroxylapatite and whitlockite are two mineral phases that can occur in several types of rocks. The possibility was taken into account even if the common phosphorus-containing rock types showing occurrence of natural whitlockite (mantle xenoliths, lunar basalts, and zoned granite pegmatites) are clearly not present in the area. This hypothesis was firmly discarded for two main reasons: the bulk density of the studied stones is about 1g/cm^3^, a value much lower than that typical of rocks (mainly between 2 and 3 g/cm^3^) and the heterogeneous and concentric microstructure observed in the studied stones is not consistent with that of most rock pebbles, which are shaped by physical transport and mechanical processes. Furthermore, tomographic, microstructural and compositional analyses confirm the biogenic origin of the stones with the mineralogical composition and the micro-texture of the Al Khiday stones clearly indicating their pathological nature.

There are four main types of biogenic stones found in the human body: gallstones (bile-duct), salivary, renal, bladder and prostate stones. These can be differentiated both by their location in the body and their mineralogical and microstructural features [1,20]. Renal stones (kidney stone, urolithiasis) form in the kidneys and can also be found in the urinary tract; they are commonly composed of calcium oxalate (CaC_2_O_4_), calcium phosphates, struvite (NH_4_MgPO_4_·6H_2_O), uric acid (C_5_H_4_N_4_O_3_), and less commonly by cystine ((SCH_2_CH(NH_2_)CO_2_H)_2_) and xanthine (C_5_H_4_N_4_O_2_). Among the urolithiasis (comprising renal, ureteral and bladder stones) bladder stones are the most common type attested archaeologically [1,3]. Gallstones, formed within the gallbladder, are characterized by variable size (from millimetres to centimetres) and composition. According to the type, they can be constituted by cholesterol crystals (C_27_H_46_O), or by bilirubin (C_33_H_36_N_4_O_6_) associated to calcium phosphate and cholesterol, or by alternated layers of cholesterol and calcium phosphate. Gallstone composition therefore is clearly at variance to the measured composition of the Al Khiday stones. Salivary stones (sialolithiasis) form within a salivary gland and are composed by a nucleus of hydroxylapatite enveloped by thin layers of whitlockite. However, it is clear the Al Khiday stones cannot be salivary stones because of their position within the skeleton.

The presence of a substantial amount of whitlockite in the Al Khiday pathological stones is a clear indication that the stones are not kidney or bladder stones. Rare intrascrotal lithiasis [20] is also reported to be composed mainly of whitlockite, though the body location and the micro-structure of the investigated stones is at variance with this interpretation.

The occurrence of ovoid micro-structures, suggestive of phosphocalcification within amylaceous bodies [21,22,23,24,25] as well as the hydroxylapatite-whitlockite association, strongly suggests that the objects are prostatic stones, although the varying proportion of hydroxylapatite and whitlockite may indicate that the whitlockite-poor stone could have formed in the upper urinary tract.

Prostatic stones develop in the prostate, a gland of the male reproductive system located in the pelvic space. More specifically, they are described as primary or endogenous when they form from prostatic fluid in acini and are composed of apatite (Ca_10_(PO_4_)_6_(OH)_2_) and whitlockite (Ca_9_(Mg,Fe)(PO_4_)_6_(PO_3_OH)) [25], with a compact nucleus and periphery of apatite layers; they can be otherwise exogenous (or secondary) and are then composed of a nucleus of uric acid or oxalate, surrounded by layers of apatite and whitlockite, or only of uric acid. They can vary in size from millimetres to more than several centimetres in diameter [25,26,27].

Reportedly, acute inflammation has an important role in the biogenesis of prostatic calculi, also being implicated in prostate carcinogenesis [27]. The contribution of different types of bacteria, including *Enterococcus faecalis*, *Staphylococcus sp* and *Escherichia coli*, to inflammation and prostatic calcifications seems to be supported by clinical studies [28,29]. These bacterial activities are also recorded in the studied stones (Fig 4E and 4F), indicating that active inflammation was present in the individual during his life. However, these imprints could also be the result of post-mortem bacterial activity, since similar features were also identified in pre-Mesolithic and Neolithic bones as non-Weld microscopic focal destruction (micro-channels and pores, up to 2 μm in diameter, surrounded by a hyper-mineralised rim, due to dissolution and re-precipitation of bone apatite during taphonomic processes) [10].

In this particular case, a more detailed study of the aetiology of such ancient calculi is prevented by the degradation of the bacterial evidence and the related organic constituents as a result of the severe post-depositional processes.

The pre-Mesolithic population of Al Khiday shows little evidence for chronic disease and can therefore be considered a fairly healthy one [30]. Stature estimation revealed tall female and male individuals, with few cases of palaeopathological injury or disease except for a high frequency of dental caries, which is unusual for a non-agricultural society [30]. Therefore, the prostatic calculi reported for Grave 188 can be considered an exceptional discovery. The effectiveness of a multi-analytical approach to the characterization of the stones has allowed us to infer their possible origin and exclude a diagnosis of renal/kidney stones or bladder stones (or even gallstones/biliary stones); types that have been more frequently recorded in antiquity [1,31,32,33]. The evidence here reported cannot reveal whether a prostatic carcinoma caused the death of this prehistoric male individual buried at Al Khiday, however, given the significant dimensions of the stones it is likely that they may have caused mechanical obstruction to the urinary tract and therefore affected the quality of his life [34]. Modern medical literature does not provide details of the symptoms this type of calculus may produce if left untreated, especially since prostatic stones are generally small, measuring only a few millimetres, and are described as asymptomatic and rather common in adults. However, prostatic stones can be associated with a reduced urinary stream, prostatism, and intense lower back and leg pain [35]. Localised symptoms, comparable to similar pathologies affecting the bladder, urethra or kidneys [36], may advance to more systemic ones causing pelvic dilatation, cystitis, renal scarring and kidney failure, potentially leading to the death of the affected individual [37] without medical intervention. It seems that individual 188 at Al Khiday reached adulthood but was affected by different pathologies during his lifetime and one of these, lithiasis, may ultimately have been responsible for his death. This pathology seems to have been an exception in this pre-Mesolithic population which demonstrates a generally good state of health, probably taking advantage of dietary resources from a rich and stable ecosystem under more favourable climatic conditions than those found in modern-day Sudan.

The archaeological and historical literature offers an intriguing description of ancient medical practices applied to patients suffering from lithiasis, illustrating the painful, and often fatal, nature of this condition [1,2,3]. Prehistoric people likely had less instruments to cope with disease or injury even if “surgical practices” are attested in the North-African Iberomaurusian Late Palaeolithic population and later on in Egypt [38,39]. Furthermore, the use of medicinal plants is known since 50000 BP Neanderthals in Europe [40]. Hence, while it is impossible to establish conclusively whether the Al Khiday individual died as a consequence of the lithiasis, in light of the touching descriptions described in literature regarding people suffering from this pathology, it is highly likely that this individual experienced extreme pain during the course of this disease, without the possibility of relief.

Future work may focus on the presence of bacteria and the possibility of their DNA analyses [41]. The three prostatic calculi may then potentially provide modern medicine with the opportunity to assess the link between this pathology and the inflammatory process started by bacteria (*E*. *coli*, *E*. *faecalis* and *Staphylococcus sp*.).

In summary, although bladder stones have been identified in other archaeological contexts, the remarkable discovery of prostatic stones from a prehistoric individual at Al Khiday is the first known example, both within the African history of disease, but also worldwide, revealing that severe prostate calcification affected men as early as 10000 BC.

## Supporting Information

S1 Fig Macro-photographs of the three stones. Optical macro-photographs of stone S1, S2 and S3 (external surface on the left and internal part on the right).
(TIF)

S2 Fig S1 μ-CT reconstruction. 3D surface reconstruction of stone S1 by digital photogrammetry.
(PDF)

S3 Fig S2 μ-CT reconstruction. 3D surface reconstruction of stone S2 by digital photogrammetry.
(PDF)

S4 Fig Secondary Mn, Fe-oxides. Optical micro-photograph of the outermost portion of stone S3 (polished section of a fragment) showing dendrites of Mn, Fe-oxides permeating the stone microstructure.
(TIF)
