# Supplementary figures and images for: Late Pleistocene/Early Holocene Evidence of Prostatic Stones at Al Khiday Cemetery, Central Sudan
Source: PLoS One. 2017 Jan 25;12(1):e0169524. doi: 10.1371/journal.pone.0169524 (PMC5266250; doi:10.1371/journal.pone.0169524)

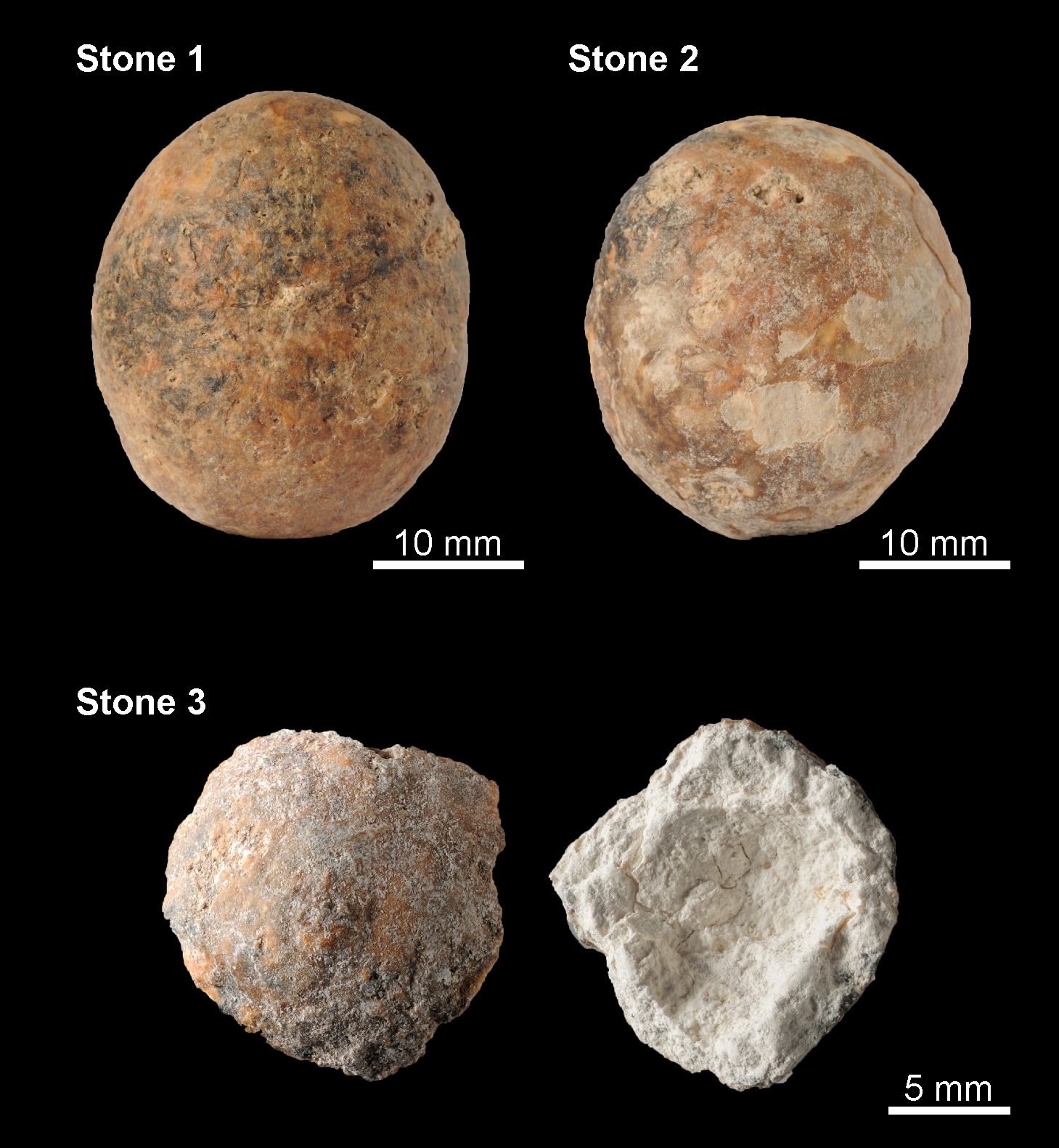

Supplement: S1 Fig — Optical macro-photographs of stone S1, S2 and S3 (external surface on the left and internal part on the right). (TIF) [file pone.0169524.s001.tif]

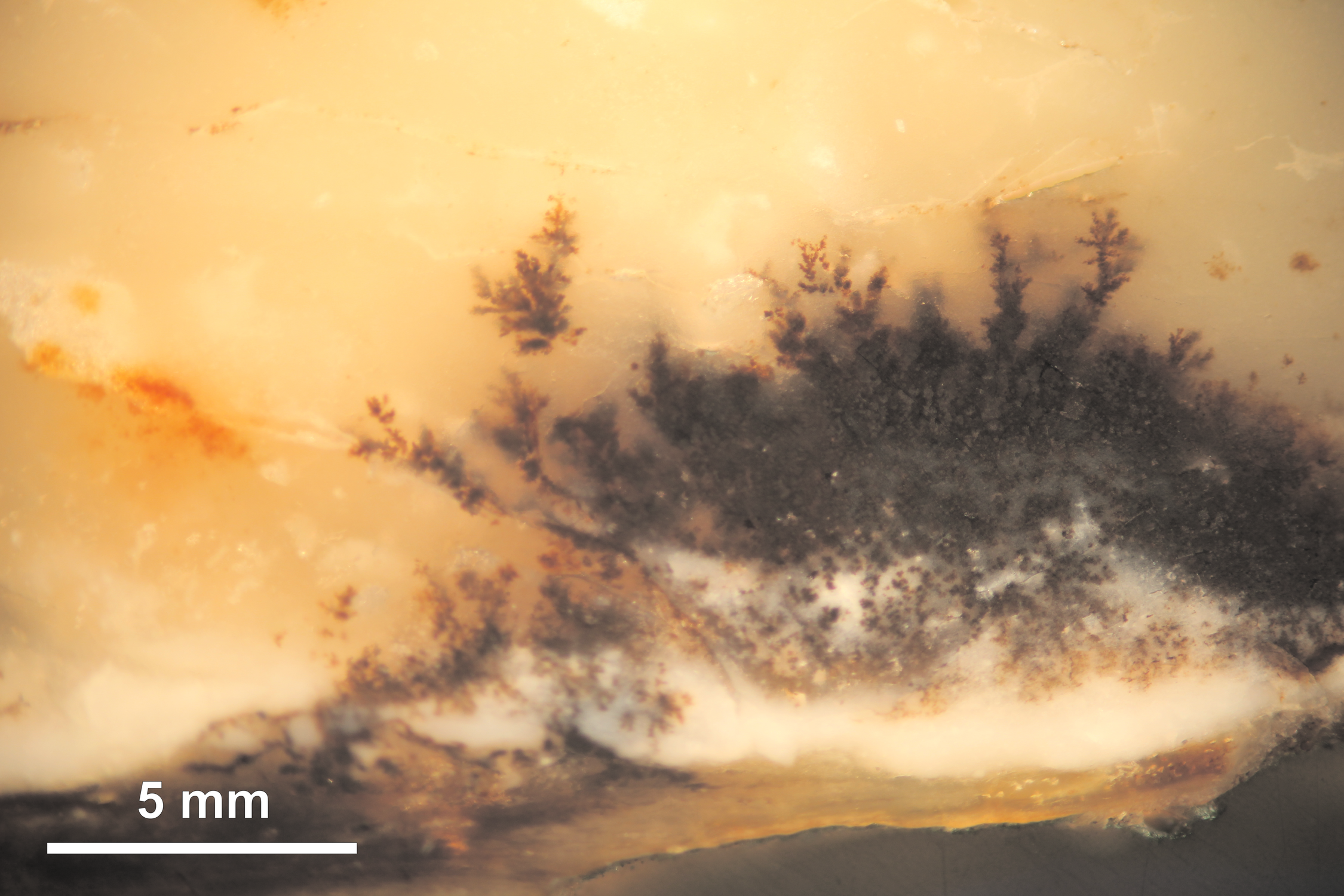

Supplement: S4 Fig — Optical micro-photograph of the outermost portion of stone S3 (polished section of a fragment) showing dendrites of Mn, Fe-oxides permeating the stone microstructure. (TIF) [file pone.0169524.s004.tif]
